# Analysis of the Multifactorial Risks of Postpartum Urinary Incontinence: A Systematic Review

**DOI:** 10.3390/healthcare14030418

**Published:** 2026-02-06

**Authors:** Nikoleta Tsinisizeli, Anastasia Bothou, Kleanthi Gourounti, Anna Deltsidou, Aikaterini Lykeridou, Giannoula Kyrkou

**Affiliations:** 1 Midwifery Department, School of Health and Care Sciences, University of West Attica, 12243 Athens, Greece; abothou@uniwa.gr (A.B.); kgourounti@uniwa.gr (K.G.); adeltsidou@uniwa.gr (A.D.); klyker@uniwa.gr (A.L.); ikirkou@uniwa.gr (G.Κ.); 2 Neonatal Intensive Care Unit, General Hospital of Nikaia “Agios Panteleimon”, 18454 Athens, Greece

**Keywords:** urinary incontinence, risk factors, predictive factors, postpartum

## Abstract

**Introduction**: Urinary incontinence (UI) is one of the most common pelvic floor disorders after childbirth and depends on hormonal changes, anatomical damage that occurs after childbirth, muscle and connective tissue weakness, fascia and nerves. UI is distinguished into three subtypes, including stress urinary incontinence (SUI), urgent urinary incontinence (UUI) and mixed urinary incontinence (MUI). **Aim**: The purpose of this review is to collect and summarize the results of studies related to the risk factors of urinary incontinence, to disseminate this information to scientists so that this major issue can be prevented, identified and managed. **Methodology**: This review followed the methodology of Preferred Reporting Items for Systematic Reviews and Meta-Analyses (PRISMA) and PECO eligibility criteria were used. We included studies published up to 2025 and not before 2019. The review was limited to studies published within the last six years in order to reflect contemporary diagnostic criteria, assessment tools and current postpartum care practices related to urinary incontinence. We searched PubMed, Google Scholar and Scopus for studies concerning the relationship between risk factors and postpartum UI. **Results**: A total of 1321 citations were identified. Following our exclusion criteria, 36 papers were selected to identify the risk factors for UI. All the research focused on the associated factors of any type of urinary incontinence. Vaginal and instrumental delivery, obesity, maternal age and the neonate’s birth weight were the main risk factors. The multiparity and incontinence symptoms before and during pregnancy were also strong risk factors. Heterogeneity across studies in assessment tools, in outcome measures and timing of postpartum assessment are some of the limitations of the study. Restriction to English-language publications and the absence of protocol registration were some of the additional limitations of the study. **Conclusions**: This problem affects the inclusion of women in society, the family, limits social activities and even their ability to work. Detection of the type of urinary incontinence by healthcare professionals, lifestyle modifications, monitoring women’s body weight and encouraging them to follow a program of pelvic floor muscle exercises should be a priority for professionals. The strategy of developing prognostic models in the coming years will be the only way to ensure the early identification and follow-up of women at high risk for urinary disorders.

## 1. Introduction

Urinary incontinence is a multifactorial health problem. In addition to pelvic organ prolapse and fecal incontinence, urinary incontinence is one of the most common pelvic floor disorders after childbirth and depends on hormonal changes, anatomical damage that occurs after birth, muscle and connective tissue weakness, fascia and nerves [1,2]. Urinary incontinence may be the result of bladder or sphincter dysfunction or both [3]. Hormonal changes during pregnancy, accompanied by weakened bladder neck support, contribute to a change in the pelvic floor support structure [4]. During that period, reduced muscle strength of the pelvic floor muscles, abnormal contractility of the urethra and relaxation of the pelvic ligaments are observed. Urinary incontinence (UI), according to the International Urinary Incontinence Society (ICS), is any involuntary loss of urine, and its severity is determined by frequency and type of leakage, confounding factors, impact on hygiene, social impact and impact on the woman’s quality of life with or without health care seeking.

Several guidelines argue that symptom reporting and self-assessment by women themselves can accurately reflect the severity of urinary incontinence symptoms without requiring measurement of the weight of the incontinence pads [5]. Some women perceive urinary incontinence as an expected symptom of childbearing, while for other women it is a social stigma. UI is distinguished into three subtypes, including stress urinary incontinence (SUI), urgent urinary incontinence (UUI) and mixed urinary incontinence (MUI) [6].

SUI is the main type of urinary incontinence, mainly in young and middle-aged women. It is caused by dysfunction of the internal urethral sphincter and pelvic floor muscles, relaxation of the fascia and ligaments and high mobility of the urethra [5]. Its appearance can be triggered by stressful situations such as childbirth and weight gain [5]. SUI is the most common form of incontinence in women of reproductive age, with an incidence of 5% to 60% worldwide [7]. It occurs after physical exertion such as running, jumping, any physical exercise, coughing, or sneezing [8,9]. It has been observed that healthy women with proper urinary continence before pregnancy will experience SUI in 15–21% [10].

Women who develop postpartum SUI have been observed to have increased bladder-neck mobility before delivery compared with those without urinary incontinence [11]. Women who experience SUI after childbirth may not return to normal function and may have permanent urinary incontinence, specifically urge urinary incontinence (UUI). In particular, studies have shown that in women who experienced urinary incontinence 3 months after childbirth, these symptoms persisted even after 6 and 12 years by 24% and 38%, respectively [12]. The study by Ferrari et al. revealed that women who performed Pelvic Floor Muscle Training (PFMT) during pregnancy rather than only in the postpartum period were at lower risk of having symptoms of SUI [13].

UUI is characterized by an urgent need to urinate that cannot be postponed, followed by an involuntary loss of urine and nocturia or frequent urination [14]. A systematic review of 22 studies showed that UUI occurs in 1.8% to 30.5% in Europe, 1.7% to 36.4% in the USA and 1.5% to 15.2% in Asia [5]. The appearance of this particular subtype is associated with age, with a greater worsening of symptoms in women near the age of 70. Its symptoms can cause intense anxiety and depression and have a negative effect on the quality of life of these women [5].

Mixed UI is the most common form of urinary incontinence that combines the two previous subtypes. Its prevalence increases with age and tends to be more common in women aged > 65 years. MUI has the greatest impact on quality of life. The symptoms tend to worsen rather than improve. Studies have reported low remission rates. Only 8% of women with mild or moderate MUI will experience complete remission after 4 years of follow-up [5].

Worldwide, rates of urinary incontinence range from 15 to 52%, with a higher incidence in women, which has a significant impact on quality of life (QOL) [15]. Studies have shown that the problem gets worse in pregnancy and is observed in 30–35% during the third trimester [16]. Changes in the neuromuscular function of the urethral sphincter during pregnancy that may persist postpartum increase the likelihood of urinary incontinence [17]. The risk of urinary incontinence after childbirth, especially in the 4–18 months after childbirth, doubles in women who experience incontinence during pregnancy [11,18].

During the postpartum period, the rates range from 7 to 12% [19]. Approximately 34.3% of women worldwide experience urinary incontinence, while 8.5% of these women need to use pads on a daily basis [20]. In relation to primiparous women, studies have shown that during the first 12 months after giving birth, 55% of primiparous women will experience urinary incontinence [21]. Some cases of urinary incontinence are temporary and the symptoms subside after three months have passed since delivery. But if these symptoms persist, the chances increase to 92% that the problem will persist 5 years later [22].

Worldwide, reported rates of urinary incontinence in women aged 20 years and older are 45% in the USA, 46% in Australia, 30.9% in China, 25% in Norway, 48% in Germany, 46% in Denmark [23], 11% in Pakistan [24], in Turkey from 20.9 to 37.11% [15], in Qatar 20.6% and in the United Arab Emirates 20.3% [3]. In France, 1 in 4 women report urinary symptoms, the severity of which depends on age, body mass index and number of deliveries [25].

The pelvic floor consists of muscles, fascia and ligaments which are responsible for supporting the pelvic organs and maintaining their proper function. Pregnancy and childbirth can cause damage to both the structure and function of the pelvic floor [9]. Hormonal and anatomical changes are observed, relaxation of the pelvic floor muscles and subsequently their reduced contractility, and the weakening of the supporting structure of the urethra as a result of pregnancy, which increases the risk of urinary incontinence after childbirth [9]. At a hormonal level, the increased concentration of estradiol during pregnancy can adversely affect the metabolic capacity of pelvic floor muscle fibers [14].

During pregnancy and the postpartum period, most women with incontinence recover their symptoms as this is not a life-threatening condition. Urinary incontinence, if it does not subside automatically after training the pelvic floor muscles, can be combined with the appearance of accompanying problems, such as embarrassment, shame, sexual dysfunction, lack of self-esteem and social isolation, which can be particularly disturbing and lead to anxiety and depression with long-term effects on the woman’s quality of life [26]. Urinary incontinence is a major problem of psychosomatic morbidity that causes substantial damage to the quality of life [27]. The scientific community tends to search for aggravating factors. These factors are divided into modifiable and non-modifiable risk factors.

Modifiable risk factors include smoking, mode of delivery, oxytocin use and prolonged second stage of labor, pregnancy weight gain, stress and obesity [27]. A large body of literature demonstrates that obesity increases the risk of urinary incontinence, but this is not an independent risk factor. For example, waist circumference and height ratio can be a strong confounding risk factor [28]. Long-term obesity weakens the pelvic floor and the supporting structures of the urethra. It has been found that every five-unit increase in a woman’s BMI increases the potential risk of UI by 20–70% [29]. Early interventions, such as weight loss and pelvic floor muscle training, could be beneficial in reducing the incidence of urinary incontinence [30].

In the case of stress, it is observed that it can be both a risk factor in the possibility of urinary incontinence and an impact on those women who face this problem [5]. In addition, pelvic floor gynecological operations, limited physical function and especially the history of pregnancies, deliveries and perineal tears tend to be important modifiable risk factors [8,15]. Deliveries that happen with the use of suction combined with episiotomy, newborn birth weight, head circumference and gestational age may increase the risk of urinary incontinence [4,19].

Some of the non-modifiable risk factors are race, age, multiparity, fetal size and medical comorbidities such as hypertension, diabetes, history of constipation, the presence of more than 2 chronic diseases and chronic cough [27]. Also, important risk factors include gender, age of the woman over 35 years, age at menopause and ethnicity [31]. Studies have shown that pregnancy, childbirth, depression and history (first-degree relatives with urinary incontinence) influence the onset and progression of SUI [5].

Dysfunction of the autonomic nervous system and changes in the level of the neurotransmitter serotonin increase the level of catecholamines and circulating cortisol, which can cause changes in the function of the urethral sphincter and can trigger the possibility of urinary incontinence [5]. Diabetes is also associated with the occurrence of urinary incontinence, as elevated blood sugar can affect the pH of the urine, making it hypertonic and can increase episodes of urinary frequency and urgency [5].

Despite the high prevalence of urinary incontinence during pregnancy and the postpartum period, and its significant impact on women’s quality of life, the available research data remain fragmented and show significant heterogeneity in terms of incidence rates, subtypes and risk factors. Although urinary incontinence is often considered transient, a significant proportion of women experience persistent symptoms in the long term, with serious psychosocial consequences. The need for a clear evidence-based understanding of the natural course and interventions, particularly in the postpartum period, necessitates the conduct of this systematic review.

## 2. Aim

The aim of this systematic review was to synthesize recent evidence on postpartum urinary incontinence, focusing on its prevalence, associated risk factors and evidence-based preventive and management approaches.

More specific objectives:

To summarize the prevalence and incidence of urinary incontinence during the postpartum period.

To identify and evaluate risk factors associated with postpartum urinary incontinence based on the available evidence.

To assess the methodological quality of the included studies using a standardized appraisal approach.

To synthesize current evidence on preventive and management interventions for postpartum urinary incontinence.

To identify gaps in the literature and highlight priorities for future research.

## 3. Methodology

### 3.1. PECO Eligibility Criteria

Population (P): Postpartum women, regardless of age, parity, or mode of delivery.

Exposure (E): Potential risk factors for urinary incontinence (e.g., mode of delivery, number of pregnancies, maternal age, BMI, obstetric complications, perineal trauma, breastfeeding, hormonal changes).

Comparison (C): Postpartum women without the same risk factors or with different levels of exposure.

Outcome (O): Incidence, prevalence, severity, duration of urinary incontinence and its impact on quality of life.

Based on this framework, the formulated research question was as follows: “What are the risk factors associated with urinary incontinence during the postpartum period, and how do they influence its incidence, severity, and impact on women’s quality of life?”

### 3.2. Study Selection

This systematic review was based on the PRISMA method (Preferred Reporting Items for Systematic Reviews and Meta-Analyses) and aggregated data published in international literature. A systematic bibliographic search was conducted across the electronic databases PubMed, Google Scholar and Scopus for studies concerning the relationship between risk factors and postpartum UI. Study selection was guided by predefined inclusion and exclusion criteria, as outlined below.

The review was limited to studies published within the last six years in order to reflect contemporary diagnostic criteria, assessment tools and current postpartum care practices related to urinary incontinence. The final search date was April 2025.

#### 3.2.1. Inclusion Criteria

Women assessed for urinary incontinence within 12 months postpartum, regardless of follow-up duration.

Studies reporting any type of urinary incontinence during the postpartum period.

Research analyzing predictive or associated risk factors for postpartum urinary incontinence.

Original research studies (cross-sectional, cohort, case–control, or longitudinal).

#### 3.2.2. Exclusion Criteria

Women with pre-existing urinary incontinence prior to pregnancy.

Studies not specifically reporting postpartum UI.

Studies including mixed populations without separate postpartum data.

Reviews, meta-analyses, case reports, letters, editorials, or conference abstracts.

Articles published in languages other than English.

### 3.3. Search Strategy

The search strategy was developed according to the PECO framework, using a combination of keywords and Boolean operators. The following terms and their variations were used:

Search String: (((postpartum women) OR (postnatal women) OR (women after childbirth)) AND ((urinary incontinence) OR (UI) OR (urine leakage))) AND ((risk factors) OR (predictive factors) OR (associated factors) OR (determinants)).

The initial search results were screened independently by two reviewers. After duplicates were removed, the remaining records were evaluated based on titles and abstracts according to the predefined inclusion criteria. Subsequently, the full texts of potentially relevant articles were assessed for eligibility. Disagreements regarding the inclusion of studies were resolved through discussion with a third reviewer to reach consensus. Only full-text articles published in English were included in the final synthesis, resulting in 36 studies selected for qualitative analysis.

### 3.4. Methodological Quality Assessment

The methodological quality and risk of bias of the studies were assessed using the NIH Quality Assessment Tool for Observational Cohort and Cross-Sectional Studies and the Mixed Methods Appraisal Tool (MMAT) for studies with mixed-methods designs, to ensure appropriate appraisal across different study types. These tools were applied to each selected study, examining specific criteria such as: sample representativeness, control of confounding factors, assurance that urinary incontinence did not pre-exist the study period, the final sample size and data adequacy. Studies were rated as low risk of bias when the majority of quality assessment domains were fulfilled and no critical methodological limitations were identified. Studies were rated as moderate risk when some domains were not fulfilled, but without substantial risk of bias affecting the validity of the results. The detailed quality ratings for each study, as derived from the application of these tools, are presented in Table 1 and Table 2.

## 4. Results

**Figure 1:**
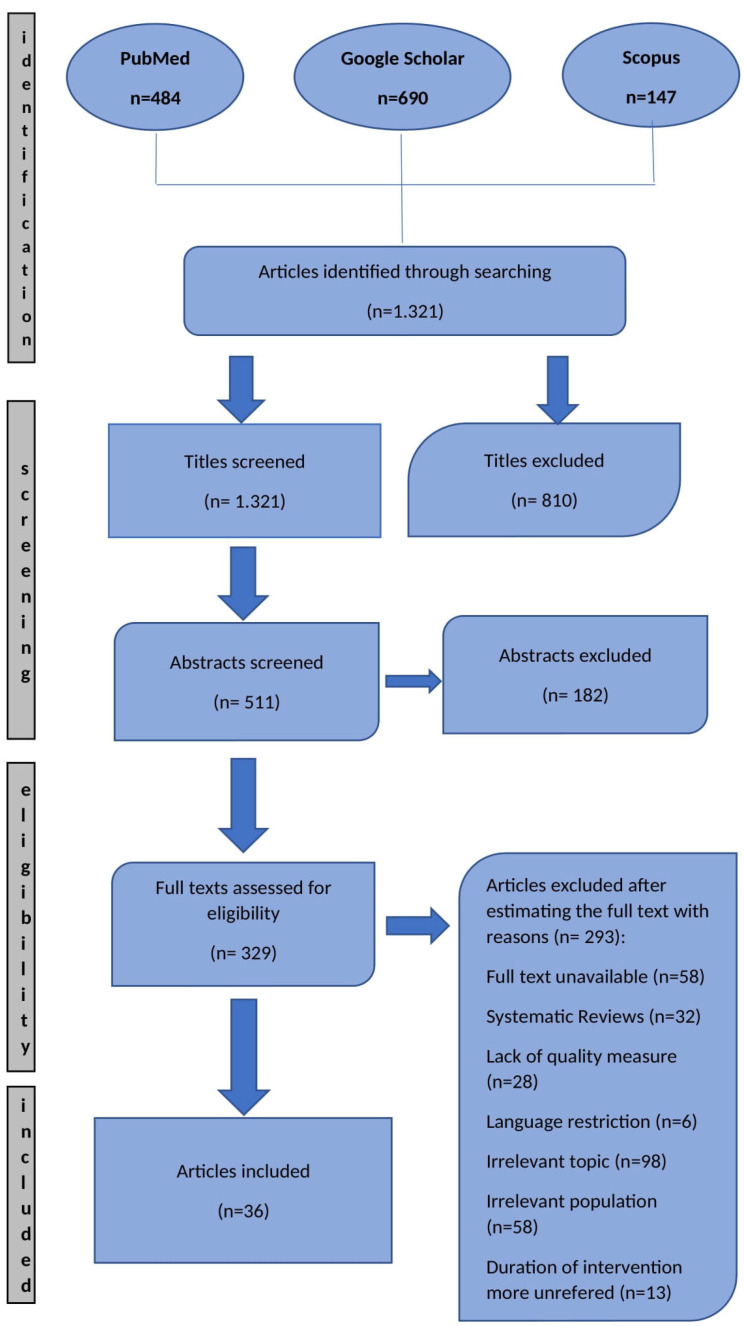
PRISMA diagram and process of articles’ selection.

In the present systematic review, the PRISMA method was used. Focusing on studies published in the last 6 years, a total of 484 articles were retrieved from the PubMed database, 690 from Google Scholar and 147 from Scopus. 1321 results were obtained. Then, by reviewing the titles and abstracts of the articles, 810 articles and 182 abstracts were excluded, respectively. Finally, 329 full texts were assessed for eligibility. After reading the full text, 293 articles were excluded. Finally, 36 articles were included in this review. Risk of bias assessment indicated variability in methodological quality across studies, with detailed judgements presented in Table 1 and Table 2. The search algorithm refers to Figure 1.

### 4.1. Characteristics of the Included Studies

Table 3 summarizes the characteristics of the studies included in this review. The study sample in each of the articles varied from 140 to24.985 women. A total of 84.721 women were enlisted in the included studies. This systematic review included 10 cross-sectional studies [3,6,8,10,25,32,33,34,35,36], 14 prospective studies [4,6,13,16,18,21,26,27,29,31,37,38,39,41], 9 retrospective studies [7,11,12,14,17,20,40,42,43], 1 cohort study [22], 1 descriptive [15] and 1 longitudinal study [1].

### 4.2. Data Collection Methods

Data on urinary incontinence were collected either through telephone interviews [10,36,38] or via self-administered questionnaires [3,6,9,11,15,18,27,32,33,39], provided to the participants. Some of the studies used ICIQ-UI SF Questionnaire [1,13,14,16,22,30,35,38,42] and two studies used PFDI-20 and ICIQ-FLUTS Questionnaire, respectively [20,21]. Three researchers, through UDI-6 and IIQ-7 questionnaires, tried to reveal the areas in the quality of life of the women that may have been influenced by the urine leakage [4,17,25,31]. Rajavuori et al. questionnaire based on the Wexner incontinence score [29].

Juraskova et al. screened for symptoms of anxiety and postpartum depression in new mothers by means of the Edinburgh Postnatal Depression Scale Questionnaire [26]. One researcher used a combined reproductive health and kidney conditions—urology questionnaire in order to cover areas such as menstrual history, pregnancy history, family history of urinary tract issues and urinary symptoms [34]. Last but not least, Liu et al. performed a clinical examination assessing the woman’s ability to contract their pelvic floor muscles using the modified Oxford grading system [7]. The assessment of predictive risk factors for urinary incontinence focused on either the intrapartum and postpartum period or the prenatal period.

### 4.3. Risk Factors Identified in Individual Studies

In the case of researchers who used telephone interviews, Zhong et al. focused on maternal age and women’s lack of knowledge, while the other two studies searched for confounding factors such as obesity and weight gain during pregnancy, history of pregestational SUI, age of the woman, type of delivery and pregestational diabetes. The duration of the follow-up was not more than one year postpartum [10,40,43].

In the case of self-administered questionnaires, Liu et al. revealed two risk factors, such as the mode of delivery and episiotomy [9]. Jansson et al. [18,39] and Patel et al. [27] also searched for additional factors such as maternal age, connective tissue deficiency, familial pelvic floor dysfunction and history of urinary tract infection. Alamri et al. focused on multiparity, maternal age and vaginal delivery [3] while Subki et al. measured the Body Mass Index and examined an important confounding factor, which was fecal incontinence [33].

### 4.4. Findings Based on Measurement Instruments

The International Consultation on Incontinence Questionnaire—Urinary Incontinence Short Form (ICIQ-UI SF) was used by 9 researchers in this review. Most of them were focused on maternal age, multiparity, family history of UI, high birth weight, BMI postpartum, vaginal delivery, especially forceps delivery [42], interdelivery interval less than 41 months [14] and episiotomy or spontaneous tears [13,16,22,35,38,42]. Diez-Itza et al. referred to the prolonged second stage of labor for more than 1 h and to the SUI during pregnancy [1]. Wang et al. assessed chronic coughing and constipation as the main risk factors for UI [30].

The Pelvic Floor Distress Inventory Questionnaire (PFDI-20) was the measurement instrument of Cheng et al., which assessed the impact that pelvic floor disorders have on quality of life in women [20]. Number of pregnancies, oxytocin use and delivery method were the main risk factors. Besides these, they mentioned three additional risk factors such as occupation, residence and educational level. ICIQ-FLUTS Questionnaire evaluated the severity and impact of female lower urinary tract symptoms on quality of life [21]. Huang et al. found that family history, perineal laceration after vaginal birth, frequent coughing and constipation were some of the risk factors that were revealed in their study. They also searched for a different risk factor, which was the consumption of coffee or tea [21].

The use of the Urinary Distress Inventory Questionnaire (UDI-6) and Incontinence Impact Questionnaire (IIQ-7) helped the researchers to estimate the quality of life in women who suffer from urine leakage [4,17,25,31]. Naorungrot et al. searched for risk factors such as diabetes mellitus, intrauterine growth restriction, stillbirth, breech presentation and multiple pregnancies [31]. Abushamma et al. referred to smoking, physical inactivity, unemployment and caffeine consumption as possible risk factors for UI [25]. Chang et al. found that maternal age, stillbirth and severe perineal lacerations were the risk factors for UI [17], while Ahlund et al. referred only to urinary leakage symptoms as a risk factor, before and during pregnancy [4].

Bonasia et al. used the National Health and Nutrition Examination Survey (NHANES), a program of the National Center for Health Statistics, to assess the health status of reproductive-age women. This was the main strength of the above study and also the assessment of urinary incontinence during the first 24 months after birth. Among the risk factors of UI were birth weight more than 4000 g and smoking [34].

### 4.5. Primary Risk Factors for Postpartum Urinary Incontinence

The results revealed that the primary risk factors for urinary incontinence during the postpartum period are vaginal delivery, obesity and maternal age. However, not only the above but also symptoms of UI before and during pregnancy, multiparity, the high birth weight of the newborn and the overall weight gain of the women during pregnancy are strong risk factors. The strengths of the study of Magnani et al. were the number of women who had been followed up for a period of over 12 to 24 months after birth. The main risk factor was the weight gain during pregnancy. The cases of UI were improved within the period of the 12th and 24th months after birth [32]. The influencing factors from the included studies are presented in Table 4.

### 4.6. Strengths and Limitations of the Included Studies

Ahlund’s study had a number of limitations that may have influenced the results, such as the fact that the study sample came only from two delivery wards of one hospital in Stockholm, and there was a lack of information regarding the women’s history before pregnancy [4]. The studies of Diez-Itza and Gabilondo were the only studies that investigated the long-term persistence of urinary incontinence from 6 months to 12 years after the first delivery. The very long follow-up was the main strength of these studies. The main limitation of the study of Diez-Itza was that it was not possible to perform a stress test in order to assess a strong risk factor, such as antenatal bladder neck mobility [1,37].

The main strength of Chang et study was the follow-up time with multiple time points, 3, 6 and 12 months after birth [41]. The study of Pang et al. had several limitations, such as the loss to follow-up of a high percentage of participants and the unmeasurement of the frequency and volume of urine leakage and its impact on the women’s quality of life [6]. Patel’s study gathered a population of white, college-educated women, which may not allow the generalization of the results to other populations [27]. The follow-up time of the included studies is presented in Table 5.

Zhong et al. included women with a history of two deliveries, so the generalizability of the results to women with greater parity was not possible [40]. The limitation of the cross-sectional study of Abushamma et al. was that there was no BMI data for all the Palestinian women owing to the unwillingness of many women to reveal their weight for scientific reasons [25]. The strength of Ferrari’s study relied on the large amount of data collected from the beginning of pregnancy till the 12th month after birth to assess the risk factors that may lead to UI during the postpartum period [13].

### 4.7. Risk Prediction Models

Huang et al. developed a risk prediction model for urinary incontinence during the postpartum period. They are based on the following parameters: urinary incontinence during pregnancy, family history of urinary incontinence, consumption of caffeine, frequent cough and last but not least mode of delivery [21]. Zhang et al. created another risk-predictive model including pre-pregnancy BMI, height, delivery mode and induction method, fetal weight, stress urinary incontinence during pregnancy and perineal condition [43]. The risk predictive models facilitate early prevention and are significant achievements in this field.

**Figure 2:**
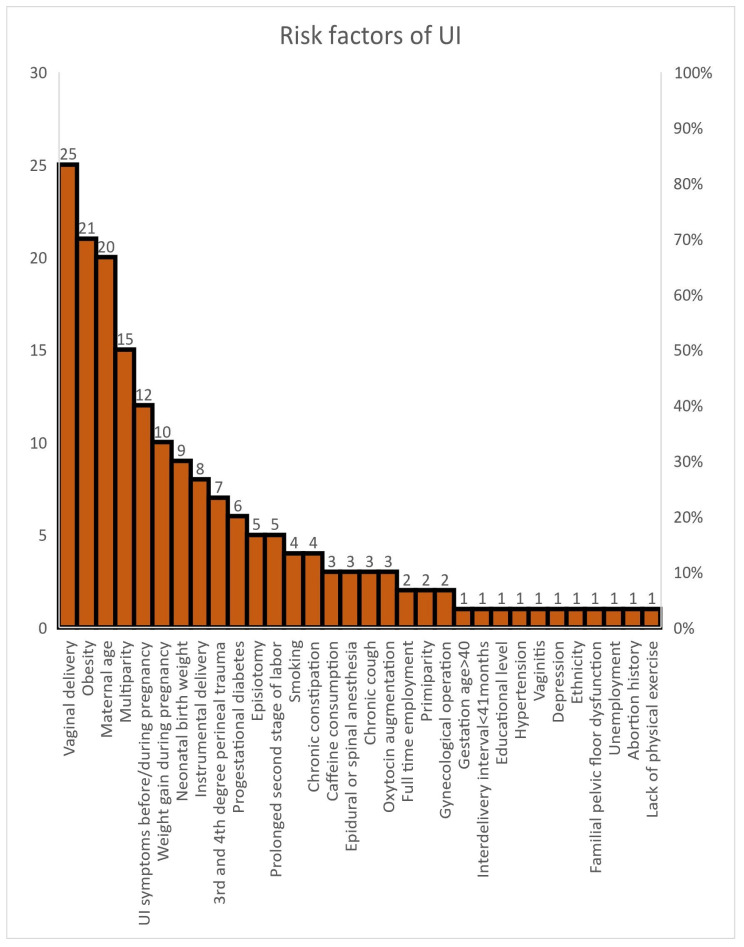
Schematic representation of risk factors of urinary incontinence.

The results from the studies are presented in Figure 2.

## 5. Discussion

### 5.1. Sociodemographic Predictors-Body Mass Index and Obesity as Risk Factors

Sociodemographic factors identified, such as age, education, BMI and unemployment, are important factors associated with postpartum urinary incontinence. Regarding increased BMI, studies suggest that it may be associated with increased pressure on the bladder and may impair blood supply to this area [2,33]. In the study by Jia et al., vaginal delivery combined with high BMI was strongly associated with urinary incontinence during the postpartum period, while the problem was more likely to resolve in women with low BMI, in those who had delivered by cesarean section and in women who had mild symptoms of urinary incontinence [38].

The study by Pang et al. shows that proper management of conditions such as obesity and diabetes may be associated with a reduction in the frequency of urinary incontinence episodes [6]. The study by Wang et al. confirms that obesity and BMI are associated with an increased risk of urinary incontinence one year postpartum [49]. Control of weight gain appears important and pregnant women should be informed about the recommended weight gain during pregnancy.

### 5.2. Mode of Delivery and Obstetric Interventions

In Siahkal’s study et al., spontaneous vaginal delivery, invasive vaginal delivery and episiotomy were associated with a higher likelihood of urinary incontinence [19]. Meta-analysis by Wang et al. showed that maternal BMI, pregnancy and vaginal delivery were associated with postpartum UI, particularly SUI, while cesarean section appeared to be associated with a lower likelihood of UI. Regarding vaginal delivery, the use of forceps was associated with higher rates of UI, whereas vacuum extraction was not associated with an increased risk [2]. Gao et al. reported that vaginal delivery, newborn’s weight, duration of the second stage of labor, increased BMI and epidural analgesia were associated with postpartum urinary incontinence [11].

### 5.3. Prevalence and Characteristics of Postpartum UI

The study by Liu et al. reported that women who delivered vaginally had a higher prevalence of SUI compared to those with cesarean section, while episiotomy was associated with increased SUI rates [7]. The study by Chang et al. demonstrated that the prevalence of SUI one year after vaginal birth was 16.5%, with a significantly higher prevalence in older primiparous women (>35 years) compared to younger primiparous women [17].

Zhong et al. reported an association between prior postpartum SUI and increased likelihood of SUI in multiparous women one year postpartum [40]. The one-year follow-up period is commonly considered appropriate, as pelvic floor recovery typically occurs within this timeframe [39].

### 5.4. History of UI and Long-Term Predictors

The longitudinal study by Pang et al. showed that spontaneous and instrumental vaginal delivery, diabetes, middle age, chronic cough, excessive gestational weight gain and smoking were associated with SUI [6]. Novo et al. reported that age is associated with urinary incontinence, potentially due to age-related changes in urethral sphincter muscle fibers [10]. According to Dias do Rego et al., home birth was associated with a higher likelihood of stress urinary incontinence compared to hospital birth [8].

Huber et al. reported that urinary incontinence four years postpartum was more prevalent among women who experienced pelvic inflammatory symptoms during the first postpartum year [50]. Moossdorf-Steinhauser et al. found that the prevalence of urinary incontinence increased over time postpartum [44]. Management strategies should be individualized based on symptom severity [5].

### 5.5. Lifestyle, Exercise and Modifiable Factors

Although pelvic floor muscle function typically improves 6–10 weeks postpartum, several studies indicate that urinary incontinence may persist for up to one year [31]. Abushamma et al. reported that lack of physical exercise was associated with higher rates of urinary incontinence [25]. Yang et al. found that vaginal delivery was the factor most strongly associated with postpartum urinary incontinence [35].

### 5.6. Pathophysiological Mechanisms

Episiotomy has been associated with alterations in pelvic floor structures, particularly connective tissue and neuromuscular stretching [22]. Postpartum pelvic floor tissues may undergo neurological and collagen-related changes, which may contribute to pelvic floor dysfunction [11]. Dai et al. reported that lateral episiotomy was associated with more severe symptoms of stress urinary incontinence [51].

### 5.7. Protective Factors

Ferrari et al. identified low body weight and cesarean section as factors associated with lower rates of UI [13]. Pizzoferrato et al. reported that cesarean section was associated with lower urethral mobility and reduced urinary symptoms [45]. However, cesarean section remains controversial, as vaginal delivery also confers maternal and neonatal benefits [46].

Zhang et al. found that epidural analgesia was associated with higher rates of postpartum SUI, possibly due to prolonged labor duration [43]. Liu et al. reported that multiparity was associated with a higher likelihood of postpartum SUI [9]. Urethral hypermobility has been linked to mechanical strain during pregnancy and vaginal delivery [39].

Patel et al. reported that previous urinary incontinence and vaginal delivery were strongly associated with postpartum UI [27]. Similarly, Wang et al. identified urinary incontinence during pregnancy, delivery mode, maternal age and gestational age as key prognostic factors associated with postpartum UI [30]. Xu et al. reported that a longer inter-delivery interval was associated with lower rates of postpartum urinary incontinence [14].

### 5.8. Socioeconomic and Psychosocial Influences

Financial difficulties, psychosocial stress and reduced social support were associated with increased urinary incontinence symptoms [13,52]. Chang et al. reported that full-time employment was associated with postpartum urinary incontinence [16]. Abushamma et al. confirmed similar findings [25].

Xue et al. identified genetic, lifestyle and socioeconomic factors associated with urinary incontinence [23].

### 5.9. Endocrinological, Biological and Quality-of-Life Factors

Hormonal changes during pregnancy may influence the development of SUI [53,54]. Genetic and microbiome alterations have been associated with urinary incontinence [21,55]. Juraskova et al. reported an association between urinary incontinence and depression [3,26].

## 6. Strengths and Limitations

This systematic review synthesizes recent evidence on postpartum urinary incontinence using a structured and transparent methodology in accordance with PRISMA guidelines. The inclusion of multiple study designs and a broad range of risk factors provides a comprehensive perspective on postpartum urinary incontinence as a multifactorial condition, while dual independent screening enhanced the robustness of study selection.

Nevertheless, heterogeneity across studies in outcome measures, assessment tools, definitions of key variables and timing of postpartum assessment should be acknowledged. Due to heterogeneity in outcome measures and assessment tools, results were synthesized narratively rather than quantitatively, and findings should therefore be interpreted with caution. Residual confounding cannot be excluded, as most included studies were observational and adjustment for all potential confounders was not uniformly possible. Limitations of the review process should also be considered, including restriction to English-language publications, selection of specific electronic databases and the potential for publication bias. The absence of protocol may further affect transparency.

## 7. Conclusions

Episodes of urinary frequency and urinary incontinence may significantly affect the quality of life of a woman, her psychology, sexual life and the way she copes with daily activities, causing socio-economic costs [44,52]. The early recognition and investigation of the risk factors associated with this dysfunction could facilitate health professionals to develop prevention and treatment strategies, emphasizing the change in obstetric care, improving the prognosis and reducing the adverse effects that these could have on a woman’s quality of life [22]. The strategy of developing prognostic models in the coming years may be the only way for the early identification and follow-up of women at high risk for urinary disorders.

Detection of the type of urinary incontinence by healthcare professionals, lifestyle modifications, monitoring women’s body weight and encouraging them to follow a program of pelvic floor muscle exercises should be a priority for professionals [41]. Strengthening the knowledge of healthcare professionals is necessary to provide targeted intervention to patients when deemed necessary. At the same time, the strengthening of awareness and the understanding of dysfunctions by the women themselves could significantly reduce long-term morbidity. Women have a right to know the possible dysfunctions they may experience from their pelvic floor, as this would allow them to control some of the modifiable risk factors that can be avoided.

## Figures and Tables

**Table 1:** NIH Quality assessment tool for observational cohort and cross-sectional studies.

Study	1. Research Question Clear? (Y)	2. Population Defined? (Y)	3. Participation Rate ≥ 50%? (Y)	4. Subjects/Criteria Uniform?	5. Sample Size Justification? (Y)	6. Exposure Before Outcome? (Y)	7. Timeframe Sufficient?	8. Examined Different Exposure Levels?	9. Exposure Measures Valid/Consistent?	10. Exposure Assessed Over Time?	11. Outcome Measures Valid/Consistent?	12. Outcome Assessors Blinded? (Y)	13. Loss to Follow-up ≤ 20%?	14. Confounding Variables Adjusted? (Y)	Quality Rating	Risk of Bias
Magnani P. (2019) [32]	Y	Y	Y	Y	Y	Y	Y	Y	Y	N	Y	Y	Y	Y	Good	Low
Subki A (2019) [33]	Y	Y	Y	Y	Y	Y	Y	Y	Y	N	Y	Y	NA	Y	Good	Low
Novo R (2020) [10]	Y	Y	Y	Y	Y	Y	Y	Y	Y	Y	Y	Y	Y	Y	Good	Low
Dias Do Rego (2021) [8]	Y	Y	Y	Y	Y	Y	Y	Y	Y	N	CD	Y	CD	Y	Good	Low
Pang H (2021) [6]	Y	Y	Y	CD	Y	Y	Y	Y	CD	CD	CD	Y	NA	Y	Good	Low
Bonasia K (2023) [34]	Y	Y	Y	Y	Y	Y	Y	Y	Y	Y	Y	Y	Y	Y	Good	Low
Yang X (2023) [35]	Y	Y	Y	Y	Y	Y	Y	NA	Y	N	Y	Y	Y	Y	Good	Low
Abushamma F (2024) [25]	Y	Y	Y	CD	Y	Y	CD	Y	Y	NR	Y	Y	NA	Y	Good	Low
Alamri A (2024) [3]	Y	Y	Y	Y	Y	Y	Y	Y	CD	Y	CD	Y	Y	Y	Good	Low
Okesina B (2024) [36]	Y	Y	Y	CD	Y	Y	Y	Y	CD	NR	CD	Y	CD	Y	Good	Low

CD: cannot determine NA: not applicable NR: not reported.

**Table 2:** Summary of the quality appraisal of studies using the MMAT (Mixed Methods Appraisal Tool).

Non-Randomized Controlled Trials	Assessment Criteria							
	Are the Participants Representative of the Target Population?	Are Measurements Appropriate Regarding Both the Outcome and Intervention (Exposure)?	Are There Complete Outcome Data?	Are the Confounders Accounted for in the Design and Analysis?	During the Study Period is the Intervention Administered as Intended?	Total Metrics	Score %	Risk of Bias
Ahlund et al. [4]	Y	Y	Y	Y	Y	5/5	100%	Low
Juraskova et al. [26]	Y	N	Y	Y	Y	4/5	80%	Moderate
Chang et al. [16]	Y	Y	Y	Y	Y	5/5	100%	Low
Gabilondo et al. [37]	Y	Y	Y	Y	Y	5/5	100%	Low
Jansson et al. [18]	Y	Y	Y	CT	Y	4/5	80%	Moderate
Jia et al. [38]	Y	Y	Y	Y	Y	5/5	100%	Low
Patel et al. [27]	Y	Y	Y	Y	Y	5/5	100%	Low
Rajavuori et al. [29]	Y	CT	Y	Y	Y	4/5	80%	Low
Chang et al. [17]	N	Y	Y	CT	Y	3/5	60%	High
Ferrari et al. [13]	Y	Y	Y	CT	Y	4/5	80%	Moderate
Huang et al. [21]	Y	Y	Y	Y	Y	5/5	100%	Low
Jansson et al. [39]	Y	Y	Y	Y	Y	5/5	100%	Low
Liu et al. [7]	Y	Y	Y	Y	Y	5/5	100%	Low
Naonrugrot et al. [31]	Y	Y	Y	Y	Y	5/5	100%	Low
Gao et al. [11]	Y	Y	N	Y	Y	4/5	80%	Moderate
Zhong et al. [40]	Y	Y	Y	Y	Y	5/5	100%	Low
Cheng et al. [20]	Y	Y	Y	Y	Y	5/5	100%	Low
Chang et al. [41]	Y	Y	Y	Y	Y	5/5	100%	Low
Liu et al. [9]	Y	Y	Y	Y	Y	5/5	100%	Low
Xu et al. [14]	Y	Y	Y	Y	Y	5/5	100%	Low
Xu et al. [42]	Y	Y	Y	Y	Y	5/5	100%	Low
Wang et al. [30]	N	Y	Y	Y	N	3/5	60%	High
Zhang et al. [43]	Y	Y	Y	Y	Y	5/5	100%	Low
Babini et al. [22]	Y	Y	Y	Y	Y	5/5	100%	Low
Baykus et al. [15]	Y	Y	N	Y	Y	4/5	80%	Moderate
Diez-Itza et al. [1]	Y	Y	Y	Y	Y	5/5	100%	Low

Y: Yes (if it met quality criterion), N: No (if it did not meet quality criterion), CT: cannot tell (if did not mention relevant information).

**Table 3:** Characteristics of the included studies.

A/A	First Author/Country/Year	Study Design/Sample Size	Intervention	Type of Method	Follow-Up Period	Exclusion Criteria	Results
1	Magnani PP/Brazil/2019 [32]	Cross-sectional study/7026	Age, weight gain, BMI, hours in labor, hours in labor before non-elective cesarean section, parity, birthweight, head circumference	Interview with a semi-structured questionnaire containing open-ended questions	12–24 months after birth	Women who presented UI before pregnancy, Women with other voiding dysfunctions	Obesity, excessive weight gain during pregnancy
2	S Subki A/Saudi Arabia/2019 [33]	Cross-sectional study/393	Route of delivery, episiotomy, laceration	A self-administered questionnaire including questions about sociodemographic characteristics and medical history	3 months after birth	Women with spinal cord injury, multiple sclerosis, muscular dystrophy, or cerebral palsy	Body Mass Index
3	Novo R/Spain/2020 [10]	Cross-sectional study/6436	Parity, type of birth, gestation time, newborn weight, prevalence of stress urinary incontinence before or during pregnancy	A computer-assisted telephone interview system and a questionnaire, including questions regarding the mother’s behaviors and practices during the study period	3 and 16 months after birth	Women who were pregnant, Women who had given birth at home and those who had not had sexual intercourse	Obesity, history of pregestational SUI, age of the woman, vaginal delivery, pregestational diabetes, BMI, weight gain during pregnancy
4	Dias do Rego A/Brazil/2021 [8]	Cross-sectional study/380	Parity, mode of delivery	King’s Health Questionnaire	>12 months after birth	Women who were pregnant, Women who had given birth less than one year before and those with anatomical impairment, urinary fistula and urinary incontinence before the study period	Age, parity, smoking, homebirth, diabetes, BMI
5	Pang H/China/2021 [6]	Cross-sectional study/24,985	Type of delivery (vaginal spontaneous delivery or instrumental delivery)	Self-developed questionnaire	Not determined	Women with severe mental or physical illness, Pregnant women	Vaginal delivery, instrumental delivery, weight gain during pregnancy, middle age, diabetes
6	Bonasia K/USA/2023 [34]	Cross-sectional study/560	Type of delivery, Birth weight of the baby	Reproductive Health Questionnaire and Kidney Conditions-Urology Questionnaire	24 months postpartum	Pregnant women	BMI > 30, prior vaginal delivery, delivery of a baby weighing > 4000 g, current smoking
7	Yang X/China/2023 [35]	Cross-sectional study/780	Vaginal delivery	ICIQ-UI-SF and UI QoL	6–8th week after delivery	History of urological surgery or obstetric surgery, Lower urinary tract infection	Family history of UI, parity and experience of vaginal delivery
8	Abushamma/Palestine/2024 [25]	Cross-sectional study/311	Type of delivery	ICIQ-UI and IIQ-7	Not determined	Lower urinary tract infection	Smoking, physical inactivity, caffeine consumption, unemployment and marital status
9	Alamri A/Saudi Arabia/2024 [3]	Cross-sectional study/176	Vaginal delivery	Self-developed questionnaire	2 weeks postpartum up to 1 year after delivery	Not referred	Age > 35 years, multiparity, high BMI, vaginal delivery
10	Okesina B/Nigeria/2024 [36]	Cross-sectional study/400	Type of delivery	ICIQ-UFS (Urgency Frequency Scale)	Not determined	Pregnant women, Women with urinary tract abnormalities	Age, parity, BMI
11	Ahlund S/Sweden/2020 [4]	Prospective study/410	Vaginal delivery	Questionnaire UDI-6 and ΙΙIQ-7	1 year after birth	Women with diabetes mellitus and pregnant women, Female genital mutilation, intrauterine growth restriction, stillbirth, breech presentation, multiple pregnancy	UI symptoms before and during pregnancy
12	J Juraskova M/Czech Republic/2020 [26]	Prospective study/3701	Mode of delivery	The Edinburgh Postnatal Depression Scale questionnaire and a self-developed questionnaire for urinary symptoms	6 weeks and 6 months after birth	Mothers with postpartum depression and urinary incontinence at 6 weeks	Multiparity, mode of delivery, high pre-pregnancy BMI, depression
13	Chang S/Taiwan/2021 [41]	Prospective study/1447	Type of vaginal delivery	ICIQ-UI SF	Late stages of pregnancy and 3–5 days, 1, 3, 6 and 12 months after childbirth	Pregnancies with fetal abnormalities	Maternal age of 30–34 years and >35 years. Vaginal delivery, vacuum extraction, forceps delivery, stress UI during pregnancy, multiparity
14	Gabilondo M/Spain/2021 [37]	Prospective study/479	Type of delivery	ICIQ-UI SF	Six months and 12 years after birth	Women who reported any kind of UI before pregnancy	Maternal age at first delivery > 30 years, BMI > 25 kg/m^2^, SUI symptoms during pregnancy, episiotomy, infant birth weight > 4000 g, oxytocin augmentation, spontaneous vaginal delivery, instrumental vaginal delivery
15	Jansson M/Sweden/2021 [39]	Prospective study/1049	Type of delivery	Self-developed questionnaire	Early pregnancy, 36 weeks of gestation, 8 weeks postpartum and at 1 year postpartum	First visit to maternity health care after 15 weeks of gestation, Insufficient knowledge of the Swedish language	Vaginal delivery
16	Jia G/China/2021 [38]	Prospective study/6370	Type of delivery	ICIQ-UI SF	6 weeks postpartum	Women with urinary tract abnormalities, Women with UI before pregnancy	Advanced age, multiparity, macrosomia, vaginal delivery and greater postpartum BMI
17	Patel K/USA/2021 [27]	Prospective study/2301	Type of delivery	Questions adopted from the Childbirth and Pelvic Symptoms Study	6, 12, 18 and 30 months postpartum	Delivery before 34 weeks of gestation	Mode of delivery, urinary incontinence before and during pregnancy, older maternal age, pre-pregnancy BMI, history of urinary tract infection and weight gain during pregnancy
18	Rajavuori A/Finland/2021 [29]	Prospective study/547	Type of delivery	Questionnaire based on Wexner incontinence score	20 weeks of pregnancy and 3 months after childbirth	Abnormal pregnancy, Twin pregnancy, Substance abusers	Primiparity, vaginal delivery, instrumental-assisted vaginal delivery
19	Chang S/China/2022 [16]	Prospective study/501	Type of delivery	ICIQ-UI SF	15 to 28 weeks of pregnancy and 1, 3, 6, 12 months after delivery	Fetal abnormality, Unwillingness to complete the questionnaire from pregnancy to the postpartum period	High body mass index, full-time employment, multiparity and previous vaginal deliveries, vaginal delivery, UI during pregnancy
20	Ferrari A/Italy/2024 [13]	Prospective study/8410	Type of delivery	ICIQ-UI SF	6 and 12 months postpartum	Women with urinary tract abnormalities, Women with UI before pregnancy	Advanced maternal age, overweight/obesity, vaginal delivery, episiotomy, high birth weight, spontaneous tears
21	Huang X/China/2024 [21]	Prospective study/1340	Type of delivery	ICIQ-FLUTS	12 weeks postpartum	Women with a critical surgical illness or severe internal disease, Women with a history of UI or urinary tract surgery	Family history, primiparous women, frequent coughing, frequent constipation, perineal lacerations during vaginal birth, consumption of coffee or tea, antenatal urinary incontinence
22	Jansson M/Sweden/2024 [18]	Prospective study/670	Type of delivery	Self-developed questionnaire	8 weeks and 1 year postpartum	Insufficient knowledge of the Swedish language	BMI > 30, maternal age > 35 years, vaginal delivery, familial pelvic floor dysfunction, connective tissue deficiency
23	Liu W/China/2024 [9]	Prospective study/225	Type of delivery	Self-developed questionnaire and perineal ultrasound	6–12 weeks after delivery	Women with diabetes, hypertension, severe cardiovascular and pulmonary diseases, a history of pelvic surgeries and pre-pregnancy urinary incontinence	Vaginal delivery, parity greater than one, maternal age > 31 years, bladder neck mobility > 1.88 cm, funnel angle > 19.5°
24	Naorungrot J/Thailand/2024 [31]	Prospective study/443	Type of delivery	Urinary Distress Inventory (UDI-6) and Incontinence Impact Questionnaire (IIQ-7)	2 days, 6 weeks, 3 and 6 months after delivery	women with pregestational diabetes mellitus, chronic hypertension, chronic obstructive pulmonary disease, previous abdominal and pelvic surgery, pre-existing urinary incontinence and the use of a drug that affects the urinary system	Caffeine consumption
25	Gao J/China/2021 [11]	Retrospective study/612	Type of delivery	Self-developed questionnaire	Not determined	Pregnant women, women with visual or hearing impairment and those unwilling to participate in the study	Vaginal delivery, BMI before pregnancy > 24, abortion history, duration of the second stage of labor > 90 min, newborn’s weight > 3000 g, epidural anesthesia, diabetes
26	Zhong R/China/2022 [40]	Retrospective study/172	Type of delivery	Telephone interview/ICIQ-SF	42 days, 4 months and 1 year postpartum	Women having a birth prior to 28 weeks of gestation, having more than 3 deliveries, telephone number error	Maternal age > 35 years, lack of knowledge
27	Cheng H/China/2022 [20]	Retrospective study/360	Type of delivery	PFDI-20	42 days after delivery	Women with symptoms of urinary incontinence before pregnancy and a history of pelvic surgery	Number of pregnancies, oxytocin use, occupation, residence, education level and delivery method
28	Chang S/Taiwan/2023 [17]	Retrospective study/303	Type of delivery	UDI-6 and IIQ-7	Second day and 12 months postpartum	Preterm deliveries before 32 weeks of gestation, presence of urinary incontinence before pregnancy, history of previous pelvic organ prolapse or anti-incontinence surgeries, renal diseases, diabetes mellitus type 2 and neurogenic diseases	Maternal age > 35 years, gestational age at birth > 40 weeks, severe perineal laceration
29	Liu X/China/2023 [7]	Retrospective study/301	Type of delivery	Clinical examination for pelvic floor muscle strength using the modified Oxford grading system	6–8 weeks postpartum	Women with urinary infection, chronic kidney disease, diabetes, chronic constipation, urine leakage, history of surgery, family history of pelvic organ prolapse or UI, unwillingness to cooperate and unable to complete the survey	Mode of delivery, history of deliveries (more than 1 delivery) and episiotomy
30	Xu C/China/2023 [14]	Retrospective study/2492	Vaginal delivery	ICIQ-UI SF	42–60 days postpartum	Women who had a preterm birth or a twin birth and women who had no available baseline data, such as height, weight and labor data	BMI < 25 kg/m^2^, Vaginal delivery, Inter-delivery interval < 41 months
31	Xu C/China/2023 [42]	Retrospective study/3051	Vaginal delivery	ICIQ-UI SF	42–100 days postpartum	Women with a cesarean section, abnormal postpartum recovery	Maternal age of delivery, parity, duration of the second stage of labor, infant birth weight and forceps delivery
32	Wang Q/China/2024 [12]	Retrospective study/5290	Mode of delivery	ICIQ-UI SF	Not defined	Not referred	Age, body mass index, vaginal delivery of large infant, number of vaginal deliveries, chronic coughing and constipation
33	Zhang D/China/2024 [43]	Retrospective study/1125	Mode of delivery	Telephone interview	6 weeks and one year postpartum	Urinary incontinence prior to pregnancy, preterm delivery, history of diabetes and pelvic surgery and renal diseases	Pre-pregnancy BMI, SUI during pregnancy, mode of delivery, mode of labor induction, fetal weight, perineal laceration and epidural analgesia
34	Babini D/China/2020 [22]	Cohort study/140	Vaginal delivery	ICIQ-UI SF	7 and 48 months after delivery	Neurological or renal disease, urogynrcologic malformation, active urinary tract infection, prior urogynrcologic surgery	Vaginal delivery, episiotomy, prolonged labor, third and fourth-degree perineal trauma and fewer than 6 appointments during pregnancy
35	Baykus N/Turkey/2020 [15]	Descriptive study/1220	Mode of delivery	Self-developed questionnaire	Not referred	Women with renal diseases	Age, ethnicity, gender, smoking, menopause, obesity, a gynecological operation, pregnancy, birth and chronic constipation
36	Diez-Itza I/Spain/2020 [1]	Longitudinal study/315	Mode of delivery	ICIQ-UI SF	6 months postpartum	Gestational age less than 37 weeks, diabetes mellitus, urogynecological surgery or malformations, neurological disorders, UI before pregnancy	Prolonged second stage of labor more than or equal to 1 h, SUI during pregnancy.

ICIQ-UI SF: International Consultation on Incontinence Questionnaire-Urinary Incontinence Short Form/ICIQ-UI QoL: International Consultation on Incontinence Questionnaire-Urinary Incontinence Quality of Life/IIQ-7: Incontinence Impact Questionnaire/ICIQ-UFS: International Consultation on Incontinence Questionnaire-Urgency Frequency Scale/UDI-6: Urogenital Distress Inventory/ICIQ-FLUTS: International Consultation on Incontinence Questionnaire-Female Lower Urinary Tract Symptoms/PFDI-20: Pelvic Floor Distress Inventory.

**Table 4:** Factors influencing postpartum urinary incontinence.

Influencing Factors	Included Studies
Vaginal delivery	19 [4,5,6,7,8,16,18,22,23,24,30,34,41,43,44,45,46,47]
Mode of delivery (instrumental/forceps/vacuum extraction)	9 [8,11,26,30,36,38,43,44,48]
Age of woman at birth > 35	15 [4,8,10,13,16,17,22,24,31,34,38,41,42,45,48]
Multiparity	15 [4,8,9,11,13,16,20,24,26,30,31,34,45,47,48]
Primiparity	1 [44]
BMI > 25 kg/m^2^	14 [4,6,7,9,13,16,17,22,24,32,34,41,45,49]
BMI < 25 kg/m^2^	1 [46]
Weight gain during pregnancy	4 [32,38,41,43]
Pre-pregnancy BMI	4 [18,26,36,38]
Baby’s weight > 3500 g	8 [7,16,17,18,24,36,45,48]
Urinary incontinence during pregnancy	7 [3,8,9,14,17,36,38]
Pregestational stress urinary incontinence	1 [20]
Duration of second stage of labor > 90 min	4 [5,14,18,48]
Severe perineal laceration (3rd and 4th degree)	5 [5,10,16,20,36]
Episiotomy	3 [5,16,20]
Epidural anasthesia	2 [18,36]
Interdelivery interval < 41 months	1 [46]
Pregestational diabetes	4 [13,18,41,43]
Oxytocin augmentation	2 [11,36]
Family history of urinary incontinence and pelvic floor dysfunction	4 [20,22,41,47]
Current smoking	4 [2,6,7,13]
Caffeine consumption	3 [2,20,37]
Frequent/chronic coughing	2 [20,45]
Chronic constipation	3 [6,20,45]
Connective tissue deficiency	1 [22]
History of urinary tract infection	1 [38]
Gynecological operation	1 [6]
Gestational age at birth > 40 weeks	1 [10]
Depression	1 [26]
Physical inactivity	1 [2]
Full—time employment	2 [9,11]
Lack of knowledge	2 [11,42]
Ethnicity	1 [6]
Bladder neck mobility > 1.88 cm and funnel angle > 19.5 cm	1 [31]

**Table 5:** Follow-up period.

A/A	Author	2 Weeks	6 Weeks	8 Weeks	3 Months	6 Months	12 Months	16 Months	18 Months	24 Months	48 Months	12 Years
1	Magnani P.						√			√		
2	Subki A.				√							
3	Novo R.				√			√				
4	Dias do Rego A.						√					
5	Pang H.	Not determined										
6	Bonasia K.									√		
7	Yang X.		√	√								
8	Abushamma	Not determined										
9	Alamri A.	√					√					
10	Okesina B.	Not determined										
11	Ahlund S.						√					
12	Juraskova M.		√			√						
13	Chang S.	√			√	√	√					
14	Gabilondo M.					√						√
15	Jansson M.			√			√					
16	Jia G.		√									
17	Patel K.					√	√	√				
18	Rajavuori A.				√							
19	Chang S.				√	√	√					
20	Ferrari A.					√	√					
21	Huang X.				√							
22	Jansson M.			√			√					
23	Liu W.		√		√							
24	Naorungrot J.		√		√	√						
25	Gao J.	Not determined										
26	Zhong R.		√		√		√					
27	Cheng H.		√									
28	Chang S.						√					
29	Liu X.		√	√								
30	Xu C.		√	√								
31	Xu C.		√		√							
32	Wang Q.	Not determined										
33	Zhang D.		√				√					
34	Babini D.		√									√
35	Baykus I.	Not determined										
36	Dies-Itza I.					√						

## Data Availability

No new data were created or analyzed in this study.
